# The complete mitochondrial genome of a gray reef shark, *Carcharhinus amblyrhynchos* (Carcharhiniformes: Carcharhinidae), from the Western Indian Ocean

**DOI:** 10.1080/23802359.2020.1827064

**Published:** 2020-10-07

**Authors:** Ela Patel, Andrea M. Bernard, Marissa Mehlrose, Sydney Harned, Kimberly A. Finnegan, Cristín K. Fitzpatrick, James S. Lea, Mahmood S. Shivji

**Affiliations:** a Save Our Seas Foundation Shark Research Center, Nova Southeastern University, Florida, USA; b Trinity Preparatory School, Florida, USA; c State Fisheries Genomics Lab, Department of Fisheries and Wildlife, Oregon State University, Oregon, USA; d Save Our Seas Foundation, Geneva, Switzerland; e Department of Zoology, University of Cambridge, Cambridge, UK

**Keywords:** Gray reef shark, mitogenome, *Carcharhinus amblyrhynchos*

## Abstract

We present the mitochondrial genome sequence of a gray reef shark, *Carcharhinus amblyrhynchos* (Bleeker 1856), a coral reef associated species. This is the first mitogenome for this species from the western Indian Ocean. The mitogenome is 16,705 bp in length, has 13 protein-coding genes, 22 tRNA genes, 2 rRNA genes and a non-coding control region, and demonstrates a gene arrangement congruent with other shark and most vertebrate species. This mitogenome provides a genomic resource for assisting with population, evolutionary and conservation studies for the gray reef shark, which is increasingly under threat from fisheries.

The gray reef shark, *Carcharhinus amblyrhynchos* (Carcharhinidae), has a broad tropical distribution ranging from the Western Indian to the Central Pacific Oceans. This large-bodied shark is commonly associated with coral reefs where it can constitute a major proportion of the high trophic-level predator biomass, and likely plays an important role in structuring reef communities (Friedlander et al. 2014; Speed et al. 2019). Stemming recent declines in reef shark species caused by overfishing requires urgent management and conservation measures, with concomitant information on the genetic dynamics of reef shark populations to guide these efforts (Heupel et al. 2019). The population genetic studies to date on *C. amblyrhynchos* have focused largely on the eastern Indo-Pacific distribution of this species, finding strong population structure across large oceanic distances (Momigliano et al. 2015, 2017; Boissin et al. 2019). Dunn et al. (2020) have recently reported mitogenome sequences from two *C. amblyrhynchos* individuals sampled in the Chagos Archipelago, mid-Indian Ocean. Here, we report the mitogenome of a *C. amblyrhynchos* shark sampled from the Seychelles archipelago in the western Indian Ocean, approximately 2000 km distant from the Chagos Archipelago across a deep ocean expanse.

The fin tissue used for genomic DNA extraction was obtained in 2013 from a female shark tagged at D’Arros Island (LAT: −5.41052°, LONG: 53.29583°) in the Republic of Seychelles. DNA was extracted using Qiagen’s Blood & Tissue Kit (QIAGEN, Inc. USA). The shark sample (NSU accession number OC-891) is stored in 100% ethanol at Nova Southeastern University, Guy Harvey Oceanographic Research Center, Florida, USA.

Five overlapping sections of the *C. amblyrhynchos* mitogenome were amplified by long PCR using previously published primer pairs and those designed from published *Carcharhinus* species mitogenomes. The PCR amplicons were pooled for library preparation with a Nextera XT DNA Sample Preparation kit (Illumina, San Diego, CA). Final whole mitogenome libraries were 2 × 250 bp paired end sequenced on an Illumina MiSeq sequencer. Reads were assembled using the “Map to Reference” feature in Geneious R9.1.8 and the two published *C. amblyrhynchos* mitogenomes (MT104515 and MT093205; Dunn et al. 2020) as reference sequences. Reads were mapped twice (once per published sequence), and resultant sequence assemblies were identical. The mitogenome was annotated using MitoAnnotator (Iwasaki et al. 2013; http://mitofish.aori.u-tokyo.ac.jp/annotation/input.html), and annotations confirmed by comparison to the two published *C. amblyrhynchos* mitogenomes. The program MUSCLE was utilzed to align our Seychelles *C. amblyrhynchos* sequence with the two Chagos Archipelago *C. amblyrhynchos* sequences (Dunn et al. 2020), 10 other *Carcharhinus* species mitogenomes and the carcharhinid shark *Triaenodon obesus* mitogenome as the outgroup (Figure 1). The program MEGA 10.1.8 (Kumar et al. 2018) was used to produce a maximum likelihood phylogeny using GTR + I + G as the best substitution model given by jModelTest v.2.1.10 (Darriba et al. 2012).

**Figure 1.**
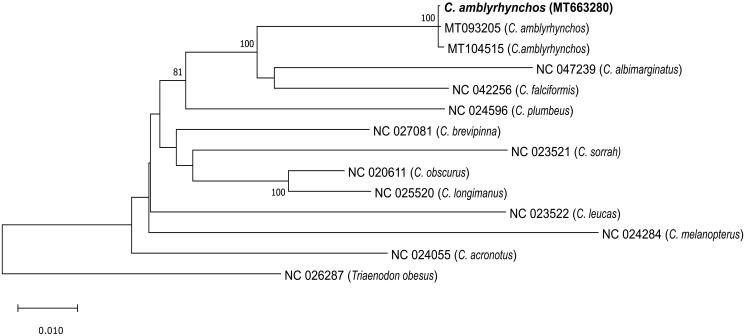
Maximum likelihood tree based on the GTR + I + G model of evolution and 1000 bootstraps. The tree with the highest log likelihood is shown. The percentage of trees in which the associated taxa clustered together is shown next to the branches for all nodes where the support is >75%. The Seychelles *C. amblyrhynchos* mitogenome (MT663280) placement is shown in bold.

The Seychelles *C. amblyrhynchos* mitogenome sequence (gb: MT663280) is 16,705 bp in length with a gene order identical to that of other sequenced *Carcharhinus* species and typical of most vertebrates, with 13 protein-coding genes, 22 tRNA genes, 2 rRNA genes, and a non-coding control region (D-loop). Nucleotide composition leaned to an A + T bias with 31.5% A, 25.2% C, 13.1% G, and 30.1% T. The ND2, ND3, ND4, COII, and CYTB genes contained incomplete stop codons. The Seychelles *C. amblyrhynchos* shark had 99.9% identity to the two Chagos *C. amblyrhynchos* individuals. The Seychelles shark contained 23 substitutions compared to Chagos individual (MT104515) sequenced by the Oxford Nanopore system, 10 substitutions compared to Chagos individual (MT093205) sequenced using the Illumina HiSeq system, and five substitutions (with two occurring in the control region) compared to both Chagos *C. amblyrhynchos* individuals. The maximum likelihood analysis is consistent with the results of Dunn et al. (2020), clustering *C. amblyrhynchos* with *C. albimarginatus* and *C. falciformis* (Figure 1).

## Data Availability

Mitogenome data supporting this study are openly available in GenBank at: https://www.ncbi.nlm.nih.gov/nuccore/MT663280. The raw Illumina sequence reads are available from the NCBI SRA database: https://www.ncbi.nlm.nih.gov/sra/SRX8934594

## References

[CIT0001] Boissin E, Thorrold SR, Braun CD, Zhou Y, Clua EE, Planes S. 2019. Contrasting global, regional and local patterns of genetic structure in gray reef shark populations from the Indo-Pacific region. Sci Rep. 9(1):15816. 31676818 10.1038/s41598-019-52221-6 PMC6825237

[CIT0002] Darriba D, Taboada GL, Doallo R, Posada D. 2012. jModelTest 2: more models, new heuristics and parallel computing. Nat Methods. 9(8):772. 10.1038/nmeth.2109 PMC4594756 22847109

[CIT0003] Dunn N, Johri S, Curnick D, Carbone C, Dinsdale EA, Chapple TK, Block BA, Savolainen V. 2020. Complete mitochondrial genome of the gray reef shark, *Carcharhinus amblyrhynchos* (Carcharhiniformes: Carcharhinidae). Mitochondrial DNA Part B. 5(3):2080–2082. 33457750 10.1080/23802359.2020.1765208 PMC7782339

[CIT0004] Friedlander AM, Caselle JE, Ballesteros E, Brown EK, Turchik A, Sala E. 2014. The real bounty: marine biodiversity in the Pitcairn Islands. PLOS One. 9(6):e100142. 24963808 10.1371/journal.pone.0100142 PMC4070931

[CIT0005] Heupel MR, Papastamatiou YP, Espinoza M, Green ME, Simpfendorfer CA. 2019. Reef shark science – key questions and future directions. Front Mar Sci. 6:12.

[CIT0006] Iwasaki W, Fukunaga T, Isagozawa R, Yamada K, Maeda Y, Satoh TP, Sado T, Mabuchi K, Takeshima H, Miya M, et al. 2013. MitoFish and MitoAnnotator: a mitochondrial genome database of fish with an accurate and automatic annotation pipeline. Mol Biol Evol. 30(11):2531–2540. 23955518 10.1093/molbev/mst141 PMC3808866

[CIT0007] Kumar S, Stecher G, Li M, Knyaz C, Tamura K. 2018. MEGA X: molecular evolutionary genetics analysis across computing platforms. Mol Biol Evol. 35(6):1547–1549. 29722887 10.1093/molbev/msy096 PMC5967553

[CIT0008] Momigliano P, Harcourt R, Robbins WD, Jaiteh V, Mahardika GN, Sembiring A, Stow A. 2017. Genetic structure and signatures of selection in grey reef sharks (*Carcharhinus amblyrhynchos*). Heredity). 119(3):142–153. 28422134 10.1038/hdy.2017.21 PMC5555095

[CIT0009] Momigliano P, Harcourt R, Robbins WD, Stow A. 2015. Connectivity in grey reef sharks (*Carcharhinus amblyrhynchos*) determined using empirical and simulated genetic data. Sci Rep. 5(1):13229. 26314287 10.1038/srep13229 PMC4551972

[CIT0010] Speed CW, Rees MJ, Cure K, Vaughan B, Meekan MG. 2019. Protection from illegal fishing and shark recovery restructures mesopredatory fish communities on a coral reef. Ecol Evol. 9(18):10553–10566. 31624567 10.1002/ece3.5575 PMC6787830

